# Impact of cariprazine on body weight and blood pressure among adults with bipolar I disorder, schizophrenia, or major depressive disorder in a real-world setting

**DOI:** 10.1186/s12991-024-00542-w

**Published:** 2025-01-27

**Authors:** Christoph U. Correll, Andrew J. Cutler, François Laliberté, Guillaume Germain, Sean D. MacKnight, Julien Boudreau, Sally W. Wade, Nadia Nabulsi, Huy-Binh Nguyen, Mousam Parikh

**Affiliations:** 1 Department of Psychiatry and Molecular Medicine, The Donald and Barbara Zucker School of Medicine at Hofstra/Northwell, Hempstead, NY USA; 2 Department of Psychiatry, Psychiatry Research, The Zucker Hillside Hospital, Northwell Health, 75-59 263rd Street, Glen Oaks, NY 11004 USA; 3 Department of Child and Adolescent Psychiatry, Charité-Universitätsmedizin Berlin, Berlin, Germany; 4 German Center for Mental Health (DZPG), Partner Site Berlin, Berlin, Germany; 5 SUNY Upstate Medical University, Lakewood Ranch, FL USA; 6 Groupe d’analyse, Ltée, Montréal, QC Canada; 7 Wade Outcomes Research and Consulting, Salt Lake City, UT USA; 8 AbbVie, North Chicago, IL USA

**Keywords:** Bipolar I disorder, Major depressive disorder, Schizophrenia, Cariprazine, Atypical antipsychotics, Weight gain, Adverse effects, Blood pressure, Real-world, Body mass index

## Abstract

**Background:**

Atypical antipsychotics are a common treatment for serious mental illness, but many are associated with adverse effects, including weight gain and cardiovascular issues, and real-world experience may differ from clinical trial data. Cariprazine has previously demonstrated a favorable safety and tolerability profile in clinical trials. Here, we evaluated the effects of cariprazine on body weight and blood pressure for bipolar I disorder (BP-I), schizophrenia, or as adjunctive treatment for major depressive disorder (MDD) using real-world data.

**Methods:**

Symphony Health’s Integrated Dataverse® with electronic medical record access (3/1/2015–10/31/2018) was used to identify adults (≥ 18 years) diagnosed with BP-I depression, BP-I mania/mixed, schizophrenia, or MDD, with ≥ 2 cariprazine dispensings (first dispensing = index) and continuous clinical activity for ≥ 12 months pre-index (baseline) and ≥ 3 months post-index. The on-treatment period spanned from index to cariprazine discontinuation, exposure to another atypical or long-acting injectable antipsychotic, or end of clinical activity/data availability. Outcomes included estimated annual linear trajectories for weight, body mass index (BMI), systolic blood pressure (SBP), and diastolic blood pressure (DBP) during baseline and on treatment. Changes were estimated using linear mixed-effects models fitted over measurements pre-index and on treatment; 95% CIs were derived from nonparametric bootstrap procedures.

**Results:**

The body weight analysis included 612 patients (BP-I, n = 331 [BP-I depression, n = 172; BP-I mania/mixed, n = 159]; schizophrenia, n = 75; MDD, n = 206). The mean patient age was 43.4 years, 75.2% were female, and the mean (SD) on-treatment period was 219 (185) days. Among patients with measurements before and during cariprazine treatment, estimated annual weight trajectories were + 3.55 (95% CI 2.38, 4.59) kg/year before cariprazine initiation and + 0.91 (− 1.17, 2.82) kg/year during cariprazine treatment. Additionally, annual linear trajectories evaluated across the on-treatment period were + 0.31 (− 0.42, 1.01) kg/m^2^/year for BMI, − 2.38 (− 4.27, − 0.76) mmHg/year for SBP, and − 0.57 (− 1.75, 0.61) mmHg/year for DBP.

**Conclusion:**

In this real-world analysis, cariprazine was associated with an estimated weight gain of + 0.91 kg/year and had minimal impact on BMI and blood pressure when evaluated up to 12 months.

**Supplementary Information:**

The online version contains supplementary material available at 10.1186/s12991-024-00542-w.

## Introduction

Individuals with serious mental illness such as bipolar I disorder (BP-I), schizophrenia, or major depressive disorder (MDD) have a life expectancy 8–28.5 years shorter than the general population [1, 2]. Approximately 60% of these deaths are due to physical illnesses, with the majority attributed to cardiovascular disease (CVD) [3]. A large-scale meta-analysis found that patients with serious mental illness have a 78% higher risk for developing CVD and an 85% higher risk of death from CVD compared to a regionally matched population [4]. Furthermore, CVD risk factors such as obesity, hypertension, type 2 diabetes, and metabolic syndrome disproportionally affect this population [5–9]. For instance, up to 68% of individuals with serious mental illness are obese and up to 61% have hypertension—further contributing to the overall disease burden of mental illness [3, 10].

Atypical antipsychotics (AAs) are a mainstay treatment option for patients with serious mental health conditions. AAs are often preferred over older generation typical antipsychotics due to their lower risk of extrapyramidal side effects [11]. However, some AAs may exacerbate CVD risk factors and metabolic abnormalities, such as weight gain, hyperlipidemia, hyperglycemia, and metabolic syndrome [6, 8, 12–15]. Because patients with serious mental illness are already vulnerable to weight gain and obesity [16], further medication-related increases in weight may lead to additional CVD risk factors, such as high blood pressure and hypertriglyceridemia [17–19]. However, AAs are a heterogeneous class of medications with different propensities for weight gain and cardiovascular effects [11, 13, 20]. For instance, although meta-analyses have shown that virtually all AAs are linked to weight gain [16], clozapine and olanzapine appear to have the highest risk. In a meta-analysis of 2- to 13-week randomized controlled trial data, clozapine and olanzapine demonstrated average weight gains of 3.01 kg and 2.73 kg, respectively [21]. Conversely, aripiprazole, cariprazine, lurasidone, and ziprasidone averaged − 0.28 kg to 0.66 kg. Weight gain due to AAs is especially concerning as treatment side effects are a predictor of nonadherence in patients with serious mental illness, especially in patients with depressive disorders [10]. Furthermore, 2 real-world, claims-based analyses found that ziprasidone was associated with an approximately 2-fold increase in the likelihood of developing hypertension compared with typical antipsychotics [22, 23], while another study found olanzapine, but not quetiapine, was associated with a significantly greater risk of hypertension compared with lithium [24]. Given this variation in adverse effects, it is critical that clinicians be aware of the specific AA metabolic profiles to minimize the occurrence of antipsychotic-induced weight gain and hypertension in their patients.

Cariprazine is a dopamine D_3_-preferring D_3_/D_2_ and serotonin 5-HT_1A_ receptor partial agonist that is Food and Drug Administration (FDA)–approved to treat schizophrenia (1.5 − 6 mg/day) and depressive (1.5 or 3 mg/day) and manic/mixed (3 − 6 mg/day) episodes associated with BP-I. Cariprazine was also approved in December 2022 as an adjunctive therapy for the treatment of MDD (1.5 or 3 mg/day). Clinical trials across indications have shown that cariprazine is associated with a weight gain of 0.54 to 1.1 kg, body mass index (BMI) change of 0.2 to 0.3 kg/m^2^, and has a minimal impact on systolic blood pressure (− 0.2 to 1.4 mmHg) and diastolic blood pressure (− 0.2 to 1.7 mmHg) [25–28]. While these clinical trials provided valuable data on weight gain, BMI, and blood pressure changes in a controlled setting, little is known about the impact of cariprazine on these outcomes in a real-world, routine care setting. To further characterize these effects, we conducted a retrospective analysis of electronic medical records from patients who were prescribed cariprazine for the treatment of BP-I depression, BP-I mania/mixed, schizophrenia, or as an adjunctive treatment for MDD. The primary objective of this analysis was to estimate and evaluate the effects of cariprazine on weight, BMI, and blood pressure in a real-world setting, with the hypothesis that the effects cariprazine on these outcomes would be similar to results from the randomized clinical trials.

## Methods

### Data source and study design

This retrospective observational study was conducted using medical/pharmacy claims data and linked electronic medical records obtained from Symphony Health, an ICON plc Company, Integrated Dataverse® (IDV) from March 1, 2015, to October 31, 2018. Symphony Health IDV is a nationally representative provider-based claims database that covers about three-quarters of the US population (or about 280 million lives annually) and permits access to in-house and third-party clinical data and linkage of these events [29]. In addition to pharmacy and medical claims data, clinical information from the linked medical records includes laboratory test results, vital signs, body measurements, and diagnoses. Data are de-identified and compliant with the Health Insurance Portability and Accountability Act (HIPAA); therefore, no institutional review board was required.

**Fig. 1:**
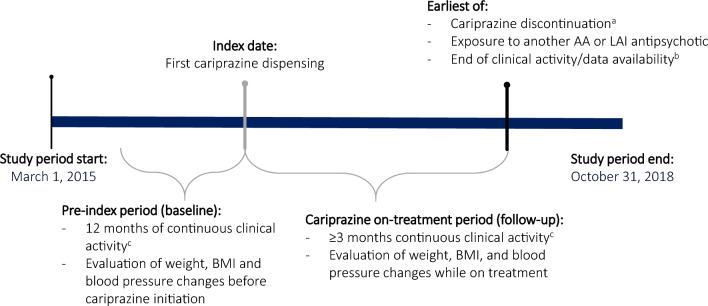
Study design. ^a^Discontinuation was defined as a ≥ 45-day gap (following a 30-day supply dispensing) or a ≥ 105-day gap (following a 90-day supply dispensing) in the days’ supply of cariprazine. ^b^Outcomes were evaluated over the full on-treatment period (truncated at 12 months) and when censored at 3 and 6 months on treatment. ^c^Continuous clinical activity was defined as consecutive quarters with ≥ 1 pharmacy, medical, or hospital claim. AA: atypical antipsychotic; BMI: body mass index; LAI: long-acting injectable

The study consisted of a 12-month pre-treatment (baseline) period before the initiation of cariprazine (index date) followed by at least 3 months of continuous clinical activity, defined as consecutive quarters with ≥ 1 pharmacy, medical, or hospital claim (Fig. 1). The cariprazine on-treatment period included the time from the index date to the earliest date of cariprazine discontinuation (defined as a ≥ 45-day gap [following a 30-day supply dispensing] or a ≥ 105-day gap [following a 90-day supply dispensing] in the days’ supply of cariprazine), exposure to another atypical or long-acting injectable (LAI) antipsychotic, end of clinical activity, or end of the study period (October 31, 2018).

### Study population

Adult patients (≥ 18 years) with at least 2 cariprazine fills (first dispensing = index date) and continuous clinical activity for at least 12 months pre-index and at least 3 months post-index were included in the analysis. Patients were required to have a diagnosis of BP-I depression, BP-I mania/mixed, schizophrenia, or MDD (Supplemental Table 1 and 2); patients with MDD must have shown evidence of adjunctive cariprazine use (off-label during this study period). Adjunctive treatment was defined as having at least 1 antidepressant therapy prescription dispensed in the 90-day pre-index period and at least 1 antidepressant therapy prescription dispensed in the 90-day post-index period with at least 14 days of overlapping supply with cariprazine. Patients with MDD who had a prior diagnosis of BP-I were excluded. Additionally, patients with a dispensing of another AA, typical antipsychotic, or a mood stabilizer/anticonvulsant on the index date or with administration of a LAI antipsychotic during the 3 months (90 days) before the index date were excluded (Supplemental Tables 3 and 4). To be included in the weight, BMI, and blood pressure analyses, patients were required to have at least 1 measurement of the corresponding outcome recorded in both the baseline and on-treatment periods; for the weight and BMI analyses, in addition to weight measurements, patients were also required to have at least 1 height measurement anytime during baseline or the on-treatment period.

**Table 1:** Baseline patient demographics and clinical characteristics

Characteristic	Analysis population^a^ (n = 612)
On-treatment period, mean (SD) [Q_1_, median, Q_3_], days	219 (185) [90, 152, 296]
Age, mean (SD), years	43.4 (13.2)
Female,^b^ n (%)	460 (75.2)
Weight, kg, mean (SD), kg	95.1 (26.9)
BMI, mean (SD), kg/m^2^	33.7 (9.1)
Quan-CCI, mean (SD)	0.69 (1.26)
Race or ethnicity, n (%)	
Black/African American	44 (7.2)
Hispanic	30 (4.9)
White	324 (52.9)
Other/Unknown^c^	14 (2.3)
Missing	200 (32.7)
Geographic region, n (%)	
Midwest	246 (40.2)
South	187 (30.6)
Northeast	123 (20.1)
West	55 (9.0)
Unknown	1 (0.2)
BMI by category, n (%)	
Underweight, BMI < 18.5 kg/m^2^	6 (1.0)
Normal, BMI 18.5 to < 25 kg/m^2^	91 (14.9)
Overweight, BMI 25 to < 30 kg/m^2^	146 (23.9)
Obese, BMI ≥ 30 kg/m^2^	369 (60.3)
Prior medication use with risk of weight gain^d^ n (%)	
Low risk of weight gain	381 (62.3)
Medium/high risk of weight gain	231 (37.7)
Comorbidities, n (%)	
Anxiety disorders	314 (51.3)
Sleep–wake disorders	175 (28.6)
Substance-related and addictive disorders	170 (27.8)
Hypertension	210 (34.3)
Diabetes	123 (20.1)
Drug abuse	99 (16.2)

BMI: body mass index; Quan-CCI: Quan-Charlson comorbidity index

^a^Reflective of the weight/BMI cohort (the largest cohort)

^b^Information on patient sex was derived from Symphony Health Integrated Dataverse records

^c^The term *other* stands for all races and ethnicities other than Black, Hispanic, and White

^d^Medications with a low risk of weight gain included aripiprazole, ziprasidone, asenapine, brexpiprazole, lurasidone, paliperidone, typical antipsychotics, or no prior atypical antipsychotic during baseline. Medications with a medium/high risk of weight gain included clozapine, olanzapine, quetiapine, risperidone, or iloperidone during baseline. Patients with medication use defined as both low risk and medium/high risk were classified as medium/high risk [32]

### Outcomes

Annual linear trajectories for weight, BMI, systolic blood pressure, and diastolic blood pressure were estimated using all measurements recorded during the pre-index (baseline) and cariprazine on-treatment period for patients across all indications combined (BP-I depression, BP-I mania/mixed, MDD, and schizophrenia). To analyze how outcomes change over the course of treatment, trajectories were also estimated using only measurements within 3 and 6 months on treatment among the subsets of patients with at least 1 measurement within 3 and 6 months on treatment, respectively.

### Statistical analysis

Descriptive statistics were used to describe baseline demographics and clinical characteristics for the analysis population. Annual linear trajectories during the baseline and on-treatment periods were estimated by using mixed-effects models fitted over all measurements pre-index and within each on-treatment time frame; results were reported using measurements within 3 months on treatment, 6 months on treatment, and across the entire on-treatment period truncated at 12 months. This model allowed the trajectory to change at the index date and accounted for repeated measurements within patients; methodology for the calculation of trajectories is shown in Supplemental Fig. 1. Patient characteristics were not included in the models because patients acted as their own controls in the pre/post design. Sample sizes varied for each time frame assessed in the study on the basis of data availability. Nonparametric bootstrap procedures with 999 replications [30] were used to calculate 95% CIs.

## Results

**Fig. 2:**
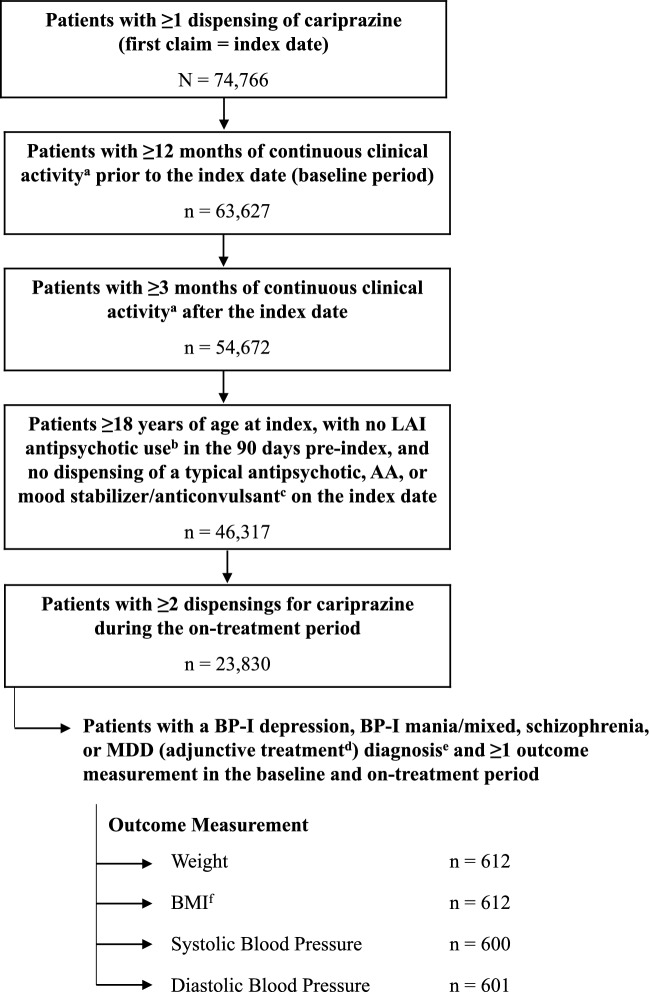
Sample selection. ^a^Continuous clinical activity is defined as consecutive quarters with ≥ 1 pharmacy, medical, or hospital claim. ^b^See Supplemental Table 4 for list of HCPCS codes for antipsychotic LAIs. ^c^See Supplemental Table 3 for list of GPI codes of AAs, typical antipsychotics, and mood stabilizers/anticonvulsants. ^d^Adjunctive treatment was defined as ≥ 1 antidepressant prescription dispensed in the 90-day pre-index period and ≥ 1 antidepressant prescription dispensed in the 90-day post-index period with ≥ 14 days of overlapping supply with cariprazine. ^e^See Supplemental Table 1 and 2 for list of *ICD-9/10* diagnosis codes. ^f^Patients with BMI analyzed also required ≥ 1 height measurement during the study period. If multiple height measurements were available, the modal value was selected; if no modal value existed, then the median value was selected. AA: atypical antipsychotic; BMI: body mass index; BP-I: bipolar I disorder; GPI: general product identifier; HCPCS: Health Care Common Procedure Coding System; *ICD: International Classification of Diseases*; LAI: long-acting injectable; MDD: major depressive disorder

Of 74,766 patients with a cariprazine dispensing, a total of 612 met the full study inclusion criteria and had baseline and on-treatment data available for weight and BMI (Fig. 2). Of these patients, 206 (34%) had a diagnosis of MDD and were using cariprazine adjunctively, 172 (28%) had a diagnosis of BP-I depression, 159 (26%) had a diagnosis of BP-I mania/mixed, and 75 (12%) had a diagnosis of schizophrenia. The mean (SD) on-treatment period for was 219 (185) days; the average patient age was 43.4 years, and 75.2% were female. Mean weight at baseline was 95.1 kg, and more than 80% of patients had a baseline BMI categorizing them as overweight (BMI of 25 to < 30 kg/m^2^) or obese (BMI ≥ 30 kg/m^2^) [31] (Table 1). Over one-third of patients had previously used a psychiatric medication with a medium or high risk of weight gain, defined as prior use of clozapine, olanzapine, quetiapine, risperidone, or iloperidone [32]. The samples with systolic and diastolic blood pressure measurements were similar, comprising 600 and 601 patients, respectively; baseline demographics and clinical characteristics among these patient subgroups can be found in Supplemental Table 5.

### Weight and body mass index

**Fig. 3:**
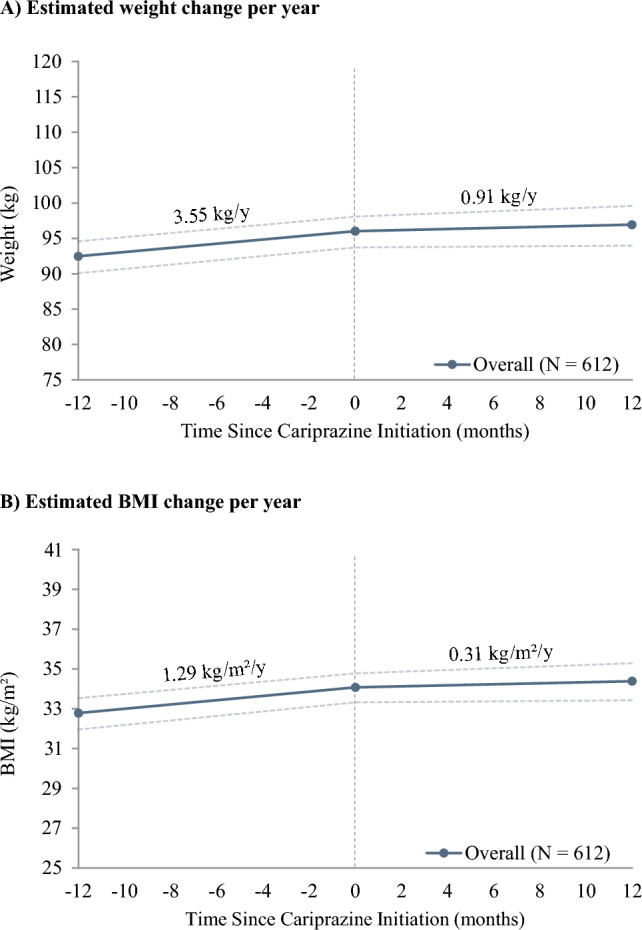
Estimated annual linear trajectories^a^ for weight and BMI during the baseline and on-treatment periods. ^a^Estimates were derived from linear mixed-effects models controlling for repeated measurements within patients; CIs, represented by dotted lines in the figures, were calculated using nonparametric bootstrap procedures with 999 replications. BMI: body mass index

Of the patients with weight and BMI data available during the baseline and on-treatment periods (n = 612), the average (SD) on-treatment period was 219 (185) days. These patients gained an estimated + 3.55 kg/year during the baseline period (estimated annual linear weight trajectory: + 3.55 [95% CI 2.38, 4.59] kg/y), which was reduced to an estimated + 0.91 kg/year after cariprazine initiation (estimated annual linear weight trajectory: + 0.91 [− 1.17, 2.82] kg/y) (Fig. 3A). The CI of the weight change trajectory during cariprazine treatment crossed zero, indicating the weight change was not significantly different from zero. Similar to the weight trajectories, the BMI of patients increased an estimated + 1.29 kg/m^2^/year during the baseline period (estimated annual linear BMI trajectory: + 1.29 [0.86, 1.66] kg/m^2^/y) and was reduced to an estimated + 0.31 kg/m^2^/year after cariprazine initiation (estimated annual linear BMI trajectory: + 0.31 [− 0.42, 1.01] kg/m^2^/y), which was also not significantly different from zero (Fig. 3B).

**Fig. 4:**
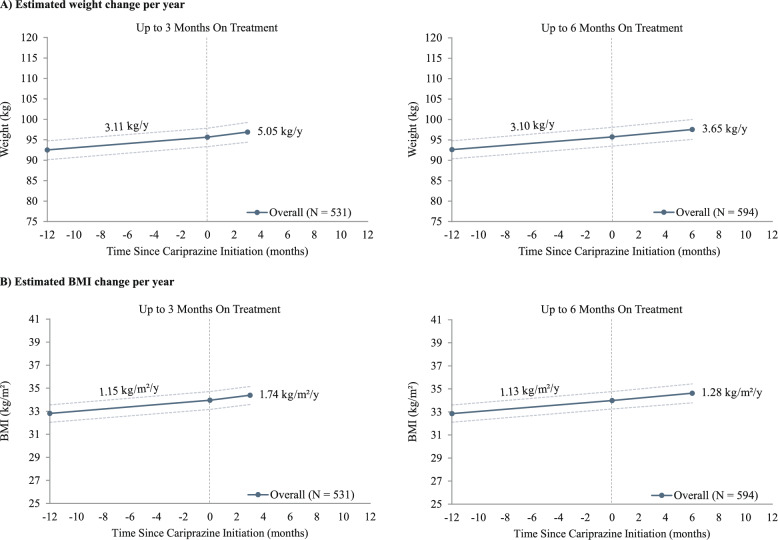
Linear trajectories^a,b^ for weight and BMI during baseline and 3 and 6 months on treatment. ^a^All available measurements were used for each time horizon, leading to different sample sizes. ^b^Estimates were derived from linear mixed-effects models controlling for repeated measurements within patients; CIs, represented by dotted lines in the figures, were calculated using nonparametric bootstrap procedures with 999 replications. BMI: body mass index

Before cariprazine initiation, the estimated annual linear weight trajectory for patients with at least 1 weight measurement up to 3 months on treatment (n = 531, 87%) was + 3.11 (1.97, 4.22) kg/y. After cariprazine initiation, the estimated annual linear weight trajectory up to 3 months on treatment was + 5.05 (1.11, 9.34) kg/y, corresponding to an average weight increase of + 1.26 kg over the first 3 months of cariprazine treatment. In patients with at least 1 weight measurement up to 6 months on treatment (n = 594, 97%), the estimated annual linear weight trajectory was + 3.10 (2.00, 4.14) kg/year prior to cariprazine initiation and + 3.65 (0.84, 6.47) kg/year after cariprazine initiation, corresponding to an average weight increase of + 1.83 kg over the first 6 months of cariprazine treatment (Fig. 4A). Estimated annual linear trajectories for BMI were + 1.15 (0.73, 1.57) kg/m^2^/year prior to cariprazine initiation for patients with at least 1 BMI measurement up to 3 months on treatment and + 1.13 (0.73, 1.53) kg/m^2^/year for patients with at least 1 BMI measurement up to 6 months on treatment. After cariprazine initiation, the estimated annual linear trajectories were + 1.74 (0.31, 3.25) kg/m^2^/year up to 3 months on treatment and + 1.28 (0.27, 2.30) kg/m^2^/year up to 6 months on treatment (Fig. 4B).

### Blood pressure

**Fig. 5:**
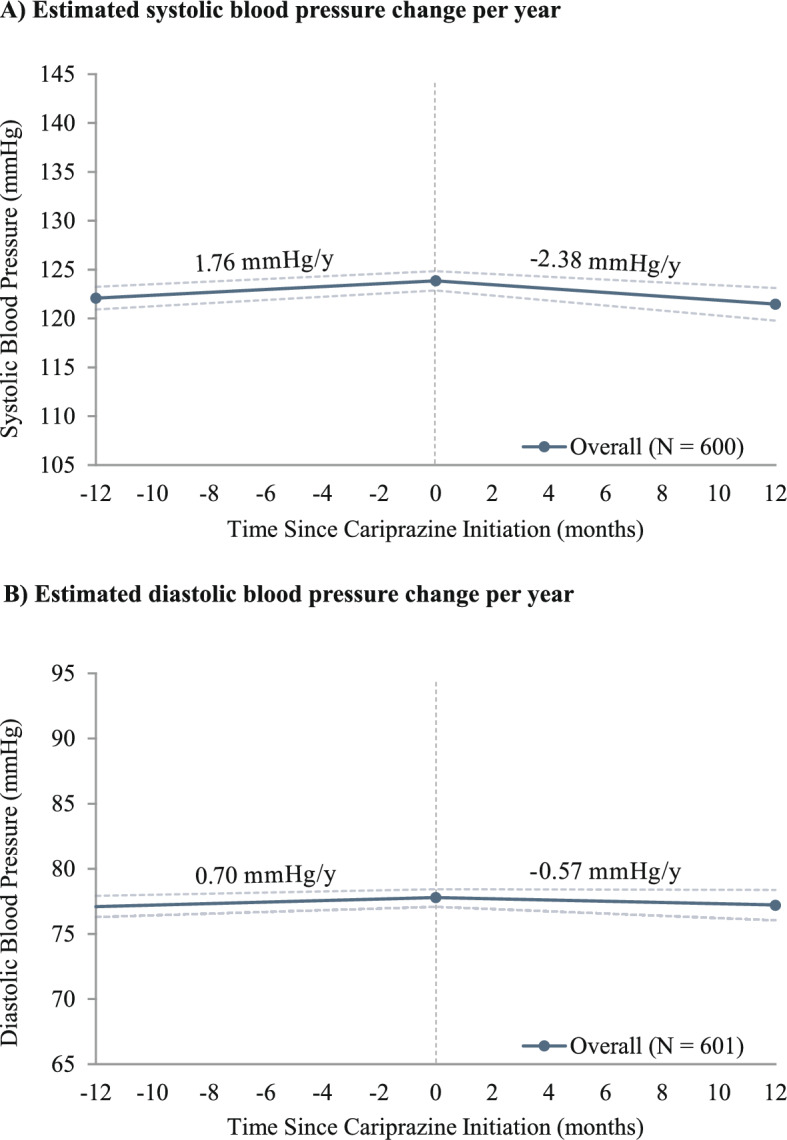
Estimated annual linear trajectories^a^ for blood pressure during the baseline and on-treatment periods. ^a^Estimates were derived from linear mixed-effects models controlling for repeated measurements within patients; CIs, represented by dotted lines in the figures, were calculated using nonparametric bootstrap procedures with 999 replications. mmHg: millimeters of mercury

**Fig. 6:**
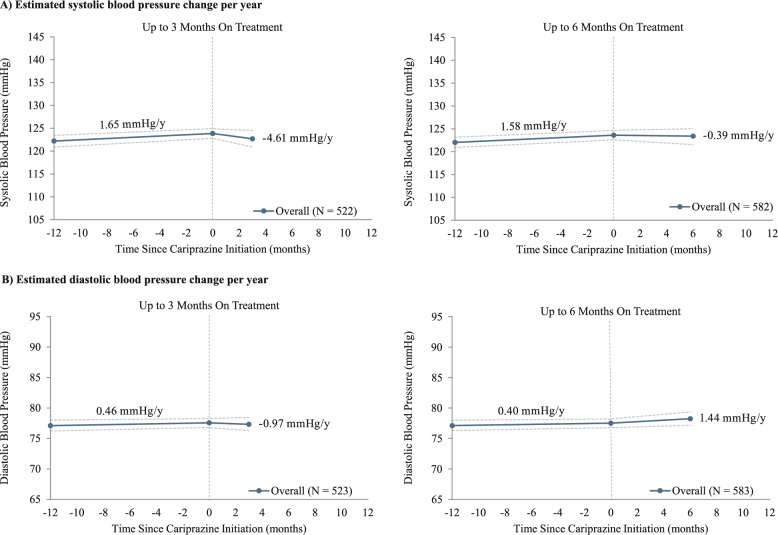
Linear trajectories^a,b^ for blood pressure during baseline and 3 and 6 months on treatment. ^a^All available measurements were used for each time horizon, leading to different sample sizes. ^b^Estimates were derived from linear mixed-effects models controlling for repeated measurements within patients; CIs, represented by dotted lines in the figures, were calculated using nonparametric bootstrap procedures with 999 replications. mmHg: millimeters of mercury

A total of 600 and 601 patients had at least 1 systolic and diastolic blood pressure measurement, respectively, during the baseline and on-treatment periods. The mean (SD) on-treatment period was 220 (186) days for the systolic cohort and 220 (185) days for the diastolic cohort. Before cariprazine initiation, the estimated annual linear trajectory for systolic blood pressure was + 1.76 (0.52, 3.04) mmHg/year, which was reduced to − 2.38 (− 4.27, − 0.76) mmHg/year after initiation of cariprazine (Fig. 5A). Diastolic blood pressure had an estimated annual trajectory of + 0.70 (− 0.29, 1.67) mmHg/year during baseline and − 0.57 (− 1.75, 0.61) mmHg/year after initiation of cariprazine, showing stable trends before and after cariprazine (Fig. 5B). Systolic blood pressure increased prior to cariprazine initiation in patients with measurements at 3 months (n = 522) and 6 months (n = 582) on treatment (+ 1.65 [0.30, 2.94] mmHg/y and + 1.58 [0.25, 2.88] mmHg/y, respectively). After cariprazine initiation, systolic blood pressure was no longer increasing at 3 months (− 4.61 [− 12.75, 3.10] mmHg/y) or 6 months (− 0.39 [− 4.08, 3.02] mmHg/y; Fig. 6A). Diastolic blood pressure changed minimally during baseline for patients with measurements at 3 months (n = 523) and 6 months (n = 583) on treatment (+ 0.46 [− 0.57, 1.54] mmHg/y and + 0.40 [− 0.71, 1.35] mmHg/y, respectively), and the trajectory was stable up to 3 and 6 months of treatment (− 0.97 [− 5.37, 3.95] mmHg/y and + 1.44 [− 0.97, 3.94] mmHg/y, respectively; Fig. 6B).

## Discussion

In our retrospective observational analysis of real-world data drawn from electronic medical records of patients prescribed cariprazine for the treatment of BP-I depression, BP-I mania/mixed, schizophrenia, or as an adjunct to antidepressant therapy for the treatment of MDD, cariprazine was associated with estimated annual linear trajectories of + 0.91 kg/year for weight, + 0.31 kg/m^2^/year for BMI, − 2.38 mmHg/year for systolic blood pressure, and − 0.57 mmHg/year for diastolic blood pressure after cariprazine initiation. Estimated annual linear trajectories of weight and BMI during the on-treatment period reflected a reduction relative to 1-year baseline trajectories. In the weight analysis, estimated on-treatment linear trajectories were higher when using measurements from within 3 and 6 months, and leveled off to + 0.91 kg/year when evaluated across the full on-treatment period. This trend is consistent with previous research showing that AA-induced weight gain often occurs early after treatment initiation before subsiding later in the course of treatment [6]. Furthermore, the average weight increase of 1.26 kg at 3 months and 1.83 kg at 6 months was consistent with clinical trial data showing an average increase of 0.6 to 1.1 kg after 6–8 weeks of cariprazine treatment [26–28]. Cariprazine initiation was also associated with estimated annual decreases in systolic blood pressure, a reduction relative to estimated annual increases during baseline, and stable diastolic blood pressure.

These findings are consistent with data from clinical trials demonstrating minimal effects of cariprazine on weight and blood pressure. In a 16-week open-label study of the safety of cariprazine in patients with manic or mixed episodes associated with BP-I, average weight gain during treatment was less than 1 kg and mean changes in systolic and diastolic blood pressure from baseline were considered small [33]. Similarly, two 48-week trials on the safety and efficacy of cariprazine for the treatment of schizophrenia found an average body weight increase of + 1.6 kg and observed that mean changes in blood pressure were generally not clinically significant [34]. Finally, an open-label trial of cariprazine for the adjunctive treatment of MDD over 26 weeks also found an average weight change of + 1.6 kg and an average systolic and diastolic blood pressure change of less than or equal to 1.0 mmHg compared with baseline [35].

Other real-world analyses have yielded comparable results to this study. Recently, Masand et al. conducted a retrospective analysis using electronic health records to evaluate the changes in weight and metabolic parameters of patients before and during treatment with cariprazine for both on- and off-label indications. Similar to our analysis, they reported that patients had an estimated weight change of − 0.8 kg/year during baseline and + 1.4 kg/year during follow-up [36]. A retrospective chart review by Greger et al. analyzing the metabolic effects of the 5 most recently FDA-approved AAs also found no significant differences in weight gain from baseline to 6 weeks, 12 weeks, and 1 year after cariprazine initiation [37]. Additionally, another retrospective chart review by Price and Price recruited patients who had directly switched from oral aripiprazole to cariprazine and evaluated the mean weight of patients during treatment with both medications. In this analysis, patients who were treated with aripiprazole for an average of 94.9 weeks had a mean weight of 90.3 kg. After being treated with cariprazine for an average of 36.7 weeks, their weight decreased to a mean weight of 83.9 kg (*P* < 0.001) [38]. These results suggest that cariprazine may have a more favorable weight profile compared with aripiprazole, although additional evidence would be needed to substantiate this hypothesis. While studies evaluating the effects of cariprazine on blood pressure are lacking, the previously mentioned real-world analysis by Greger et al. found no significant difference in systolic or diastolic blood pressure changes in patients prescribed cariprazine 6 weeks, 12 weeks, and 1 year after cariprazine initiation [37]. Because of the high prevalence of CVD risk factors and metabolic disease in patients with serious mental illness [5, 8], the minimal impact on weight gain and blood pressure of cariprazine observed in our analysis as well as in previous studies may be beneficial for this population.

Variation in the degree of weight gain associated with individual AAs may be due to different receptor binding profiles. For instance, previous meta-analyses have found that while almost all AAs cause weight gain, the magnitude of weight gain varies across different agents in the class [16, 39, 40]. Antipsychotic-induced weight gain may associate with affinity for H_1_-histamine and 5-HT_2C_ receptors, as well as muscarinic acetylcholine receptors M1 and M3, resulting in increased appetite and food intake [16, 41]. High affinity for H_1_-histamine and 5-HT_2C_ receptors also predicts diabetes risk [42]. Clozapine and olanzapine, which have high H_1_ and 5-HT_2C_ affinity [39], correspondingly carry the greatest risk for weight gain among the AAs and a high risk for glucose dysfunction [43, 44]. In contrast, cariprazine displays modest affinity for 5-HT_2C_ and H_1_-histamine receptors and no affinity for muscarinic cholinergic receptors [45]; therefore, the receptor binding affinity of cariprazine may associate with the more neutral metabolic profile seen in this real-world analysis and in previous clinical trials.

Analyses using data from electronic medical records are beneficial in showing the real-world effects of treatment. However, results should be interpreted within the context of limitations attendant to retrospective, observational analyses including these types of data. First, because this analysis included indications approved for cariprazine treatment (BP-I mania/mixed, BP-I depression, schizophrenia) and indications that were off-label at the time of the analysis (adjunctive treatment of MDD), it is possible that weight and metabolic outcome trajectories vary between the indication cohorts, and patients selected for early off-label use may differ from patients prescribed cariprazine on-label today. Future analyses should examine the indication-specific effect of cariprazine on weight, BMI, and blood pressure. Second, data reflect prescriptions dispensed from the pharmacy; it was unknown whether patients took their medications or took them as prescribed. Third, due to lack of recorded monitoring of cardiometabolic parameters during the pre-cariprazine and cariprazine on-treatment time periods, only a relatively modest sample size could be included in these analyses focusing on cardiometabolic risk trajectories before and during cariprazine treatment. Fourth, the analyzable data did not include blood glucose, insulin, hemoglobin A1C, or lipid levels, which also are relevant to evaluate cardiometabolic burden. However, body weight and body weight changes are generally good proxy measures of these related metabolic parameters [13, 15, 20]. Fifth, because this was a retrospective, observational analysis, no causal links can be drawn between cariprazine treatment and observed effects because many external factors, such as the psychiatric condition itself, medication use and related cardiometabolic effects, or lifestyle, psychosocial, and socioeconomic factors, may have influenced outcomes. Future research on how previous treatments and other patient characteristics impact weight and metabolic outcomes is warranted. Sixth, patients who discontinued cariprazine early may also have had a different outcome trajectory compared with those who received cariprazine for longer periods of time or could have discontinued cariprazine due to weight gain. Finally, linear regression analyses assume that the trajectories for weight, BMI, and blood pressure were linear, and measurements were observed more frequently early in the follow-up period than later, which may reflect clinicians monitoring patients more closely after treatment initiation.

## Conclusions

In this analysis of real-world data using electronic medical records, cariprazine was associated with an estimated annual weight gain of less than 1 kg/year and a BMI change of less than 0.5 kg/m^2^/year while patients were on cariprazine treatment. Furthermore, the estimated systolic blood pressure trajectory decreased during the on-treatment period, and the estimated diastolic blood pressure trajectory during the on-treatment period was stable. These results suggest that cariprazine has generally minimal effects on weight and blood pressure in patients with a diagnosis of BP-I depression, BP-I mania/mixed, schizophrenia, or MDD. These results complement findings from randomized clinical trial data for cariprazine and can aid clinicians in making a treatment choice for their patients with serious mental illness.

## Supplementary Information

Supplementary Material 1.

## Data Availability

Data included in this analysis are available from Symphony Health Integrated Dataverse. Restrictions apply to the use of this data, which were used under license for the current study. Study data are available from the authors upon reasonable request and with permission from Symphony Health.

## Abbreviations

AA: Atypical antipsychotic
BMI: Body mass index
BP-I: Bipolar I disorder
CCI: Charlson Comorbidity Index
CVD: Cardiovascular disease
DBP: Diastolic blood pressure
FDA: Food and Drug Administration
GPI: General product identifier
HCPCS: Health Care Common Procedure Coding System
ICD: *International Classification of Diseases*
LAI: Long-acting injectable
MDD: Major depressive disorder
SBP: Systolic blood pressure
