# Supplementary material for: Impact of cariprazine on body weight and blood pressure among adults with bipolar I disorder, schizophrenia, or major depressive disorder in a real-world setting
Source: Ann Gen Psychiatry. 2025 Jan 27;24:5. doi: 10.1186/s12991-024-00542-w (PMC11773801; doi:10.1186/s12991-024-00542-w)
Supplement: Supplementary file 1 — Supplementary Material 1. [file 12991_2024_542_MOESM1_ESM.pdf]

SUPPLEMENTARY MATERIAL

Impact of cariprazine on body weight and blood pressure among adults with bipolar I disorder, schizophrenia, or major depressive disorder in a real-world setting

Christoph U. Correll, Andrew J. Cutler, François Laliberté, Guillaume Germain, Sean D. MacKnight, Julien Boudreau, Sally W. Wade, Nadia Nabulsi, Huy-Binh Nguyen, and Mousam Parikh

Supplemental Fig. 1 Model specification and visualization

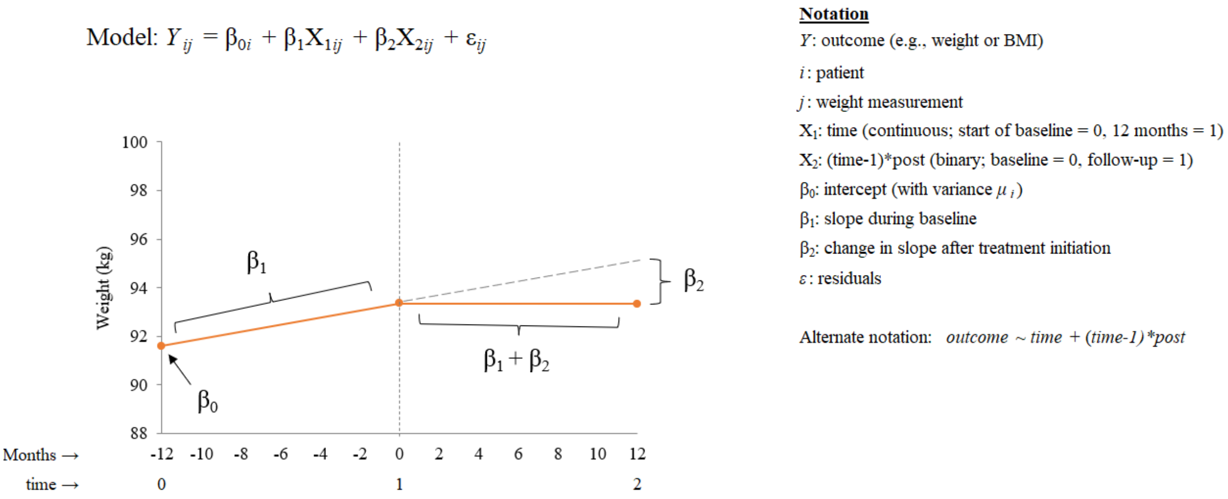

BMI, body mass index.

Supplemental Table 1 List of *ICD-10-CM* diagnosis codes for indications of interest

ICD-10-CM	**Description**
F20	*Schizophrenia*
F20.0	Paranoid schizophrenia
F20.1	Disorganized schizophrenia
F20.2	Catatonic schizophrenia
F20.3	Undifferentiated schizophrenia
F20.5	Residual schizophrenia
F20.8	*Other schizophrenia*
F20.89	Other schizophrenia
F20.9	Schizophrenia, unspecified
F30	*Manic episode*
F30.1	*Manic episode without psychotic symptoms*
F30.10	Manic episode without psychotic symptoms, unspecified
F30.11	Manic episode without psychotic symptoms, mild
F30.12	Manic episode without psychotic symptoms, moderate
F30.13	Manic episode, severe, without psychotic symptoms
F30.2	Manic episode, severe with psychotic symptoms
F30.3	Manic episode in partial remission
F30.4	Manic episode in full remission
F31	*Bipolar disorder*
F31.0	Bipolar disorder, current episode hypomanic
F31.1	*Bipolar disorder, current episode manic without psychotic features*
F31.10	Bipolar disorder, current episode manic without psychotic features, unspecified
F31.11	Bipolar disorder, current episode manic without psychotic features, mild
F31.12	Bipolar disorder, current episode manic without psychotic features, moderate
F31.13	Bipolar disorder, current episode manic without psychotic features, severe
F31.2	*Bipolar disorder, current episode manic severe with psychotic features*
F31.3	*Bipolar disorder, current episode depressed, mild or moderate severity*
F31.30	Bipolar disorder, current episode depressed, mild or moderate severity, unspecified
F31.31	Bipolar disorder, current episode depressed, mild
F31.32	Bipolar disorder, current episode depressed, moderate
F31.4	*Bipolar disorder, current episode depressed, severe, without psychotic features*

F31.5	*Bipolar disorder, current episode depressed, severe, with psychotic features*
F31.6	*Bipolar disorder, current episode mixed*
F31.60	Bipolar disorder, current episode mixed, unspecified
F31.61	Bipolar disorder, current episode mixed, mild
F31.62	Bipolar disorder, current episode mixed, moderate
F31.63	Bipolar disorder, current episode mixed, severe, without psychotic features
F31.64	Bipolar disorder, current episode mixed, severe, with psychotic features
F31.7	*Bipolar disorder, currently in remission*
F31.71	Bipolar disorder, in partial remission, most recent episode hypomanic
F31.72	Bipolar disorder, in full remission, most recent episode hypomanic
F31.73	Bipolar disorder, in partial remission, most recent episode manic
F31.74	Bipolar disorder, in full remission, most recent episode manic
F31.75	Bipolar disorder, in partial remission, most recent episode depressed
F31.76	Bipolar disorder, in full remission, most recent episode depressed
F31.77	Bipolar disorder, in partial remission, most recent episode mixed
F31.78	Bipolar disorder, in full remission, most recent episode mixed
F32	*Major depressive disorder, single episode*
F32.0	Major depressive disorder, single episode, mild
F32.1	Major depressive disorder, single episode, moderate
F32.2	Major depressive disorder, single episode, severe without psychotic features
F32.3	Major depressive disorder, single episode, severe with psychotic features
F32.4	Major depressive disorder, single episode, in partial remission
F32.5	Major depressive disorder, single episode, in full remission
F32.9	Major depressive disorder, single episode, unspecified
F33	*Major depressive disorder, recurrent*
F33.0	Major depressive disorder, recurrent, mild
F33.1	Major depressive disorder, recurrent, moderate
F33.2	Major depressive disorder, recurrent severe without psychotic features
F33.3	Major depressive disorder, recurrent, severe with psychotic symptoms
F33.4	*Major depressive disorder, recurrent, in remission*
F33.40	Major depressive disorder, recurrent, in remission, unspecified
F33.41	Major depressive disorder, recurrent, in partial remission
F33.42	Major depressive disorder, recurrent, in full remission

F33.9	*Major depressive disorder, recurrent, unspecified*

ICD-10-CM, International Classification of Diseases, Tenth Revision, Clinical Codification.

Supplemental Table 2 List of *ICD-9-CM* diagnosis codes for indications of interest

ICD-9-CM	**Description**
295	*Schizophrenic disorders*
295.00	Simple type schizophrenia, unspecified
295.01	Simple type schizophrenia, subchronic
295.02	Simple type schizophrenia, chronic
295.03	Simple type schizophrenia, subchronic with acute exacerbation
295.04	Simple type schizophrenia, chronic with acute exacerbation
295.05	Simple type schizophrenia, in remission
295.10	Disorganized type schizophrenia, unspecified
295.11	Disorganized type schizophrenia, subchronic
295.12	Disorganized type schizophrenia, chronic
295.13	Disorganized type schizophrenia, subchronic with acute exacerbation
295.14	Disorganized type schizophrenia, chronic with acute exacerbation
295.15	Disorganized type schizophrenia, in remission
295.20	Catatonic type schizophrenia, unspecified
295.21	Catatonic type schizophrenia, subchronic
295.22	Catatonic type schizophrenia, chronic
295.23	Catatonic type schizophrenia, subchronic with acute exacerbation
295.24	Catatonic type schizophrenia, chronic with acute exacerbation
295.25	Catatonic type schizophrenia, in remission
295.30	Paranoid type schizophrenia, unspecified
295.31	Paranoid type schizophrenia, subchronic
295.32	Paranoid type schizophrenia, chronic
295.33	Paranoid type schizophrenia, subchronic with acute exacerbation
295.34	Paranoid type schizophrenia, chronic with acute exacerbation
295.35	Paranoid type schizophrenia, in remission
295.50	Latent schizophrenia, unspecified
295.51	Latent schizophrenia, subchronic
295.52	Latent schizophrenia, chronic
295.53	Latent schizophrenia, subchronic with acute exacerbation
295.54	Latent schizophrenia, chronic with acute exacerbation
295.55	Latent schizophrenia, in remission

295.60	Schizophrenic disorders, residual type, unspecified
295.61	Schizophrenic disorders, residual type, subchronic
295.62	Schizophrenic disorders, residual type, chronic
295.63	Schizophrenic disorders, residual type, subchronic with acute exacerbation
295.64	Schizophrenic disorders, residual type, chronic with acute exacerbation
295.65	Schizophrenic disorders, residual type, in remission
295.80	Other specified types of schizophrenia, unspecified
295.81	Other specified types of schizophrenia, subchronic
295.82	Other specified types of schizophrenia, chronic
295.83	Other specified types of schizophrenia, subchronic with acute exacerbation
295.84	Other specified types of schizophrenia, chronic with acute exacerbation
295.85	Other specified types of schizophrenia, in remission
295.90	Unspecified schizophrenia, unspecified
295.91	Unspecified schizophrenia, subchronic
295.92	Unspecified schizophrenia, chronic
295.93	Unspecified schizophrenia, subchronic with acute exacerbation
295.94	Unspecified schizophrenia, chronic with acute exacerbation
295.95	Unspecified schizophrenia, in remission
296	*Episodic mood disorders*
296.0	
296.00	Bipolar I disorder, single manic episode, unspecified
296.01	Bipolar I disorder, single manic episode, mild
296.02	Bipolar I disorder, single manic episode, moderate
296.03	Bipolar I disorder, single manic episode, severe, without mention of psychotic behavior
296.04	Bipolar I disorder, single manic episode, severe, specified as with psychotic behavior
296.05	Bipolar I disorder, single manic episode, in partial or unspecified remission
296.06	Bipolar I disorder, single manic episode, in full remission
296.1	
296.10	Manic affective disorder, recurrent episode, unspecified
296.11	Manic affective disorder, recurrent episode, mild
296.12	Manic affective disorder, recurrent episode, moderate
296.13	Manic affective disorder, recurrent episode, severe, without mention of psychotic behavior
296.14	Manic affective disorder, recurrent episode, severe, specified as with psychotic behavior

296.15	Manic affective disorder, recurrent episode, in partial or unspecified remission
296.16	Manic affective disorder, recurrent episode, in full remission
296.4	
296.40	Bipolar I disorder, most recent episode (or current) manic, unspecified
296.41	Bipolar I disorder, most recent episode (or current) manic, mild
296.42	Bipolar I disorder, most recent episode (or current) manic, moderate
296.43	Bipolar I disorder, most recent episode (or current) manic, severe, without mention of psychotic behavior
296.44	Bipolar I disorder, most recent episode (or current) manic, severe, specified as with psychotic behavior
296.45	Bipolar I disorder, most recent episode (or current) manic, in partial or unspecified remission
296.46	Bipolar I disorder, most recent episode (or current) manic, in full remission
296.5	
296.50	Bipolar I disorder, most recent episode (or current) depressed, unspecified
296.51	Bipolar I disorder, most recent episode (or current) depressed, mild
296.52	Bipolar I disorder, most recent episode (or current) depressed, moderate
296.53	Bipolar I disorder, most recent episode (or current) depressed, severe, without mention of psychotic behavior
296.54	Bipolar I disorder, most recent episode (or current) depressed, severe, specified as with psychotic behavior
296.55	Bipolar I disorder, most recent episode (or current) depressed, in partial or unspecified remission
296.56	Bipolar I disorder, most recent episode (or current) depressed, in full remission
296.6	
296.60	Bipolar I disorder, most recent episode (or current) mixed, unspecified
296.61	Bipolar I disorder, most recent episode (or current) mixed, mild
296.62	Bipolar I disorder, most recent episode (or current) mixed, moderate
296.63	Bipolar I disorder, most recent episode (or current) mixed, severe, without mention of psychotic behavior
296.64	Bipolar I disorder, most recent episode (or current) mixed, severe, specified as with psychotic behavior
296.65	Bipolar I disorder, most recent episode (or current) mixed, in partial or unspecified remission
296.66	Bipolar I disorder, most recent episode (or current) mixed, in full remission

296.7	Bipolar I disorder, most recent episode (or current) unspecified
296.2	
296.20	Major depressive affective disorder, single episode, unspecified
296.21	Major depressive affective disorder, single episode, mild
296.22	Major depressive affective disorder, single episode, moderate
296.23	Major depressive affective disorder, single episode, severe, without mention of psychotic behavior
296.24	Major depressive affective disorder, single episode, severe, specified as with psychotic behavior
296.25	Major depressive affective disorder, single episode, in partial or unspecified remission
296.26	Major depressive affective disorder, single episode, in full remission
296.3	
296.30	Major depressive affective disorder, recurrent episode, unspecified
296.31	Major depressive affective disorder, recurrent episode, mild
296.32	Major depressive affective disorder, recurrent episode, moderate
296.33	Major depressive affective disorder, recurrent episode, severe, without mention of psychotic behavior
296.34	Major depressive affective disorder, recurrent episode, severe, specified as with psychotic behavior
296.35	Major depressive affective disorder, recurrent episode, in partial or unspecified remission
296.36	Major depressive affective disorder, recurrent episode, in full remission

ICD-9-CM, International Classification of Diseases, Ninth Revision, Clinical Modification.

Supplemental Table 3 List of GPI codes for atypical antipsychotics, typical antipsychotics, and anticonvulsants/mood stabilizers

GPI	Agent
59250015	Aripiprazole
59155015	Asenapine
59250020	Brexpiprazole
59400018	Cariprazine
59152020	Clozapine
59070035	Iloperidone
59400023	Lurasidone
59157060	Olanzapine
59070050	Paliperidone
59153070	Quetiapine
59070070	Risperidone
59400085	Ziprasidone
59200010	Acetophenazine
59200015	Chlorpromazine
59300010	Chlorprothixene
59200025	Fluphenazine
59100010	Haloperidol
59154020	Loxapine
59200045, 62994002	Perphenazine
49109902, 59200055	Prochlorperazine
59300020	Thiothixene
59200085	Trifluoperazine
59200090	Triflupromazine
72996002, 62549904, 62549903, 72600030, 75990002, 62540030	Gabapentin
62560030	Gabapentin Enacarbil
72200013, 72200020, 72200030, 72609902	Phenytoin
72170070	Tiagabine
61209902, 72600075	Topiramate
59400015, 72600020	Carbamazepine

67300030, 72500010	Divalproex
72600040	Lamotrigine
59500010	Lithium
72600046	Oxcarbazepine
72500020, 72500030	Valproate

GPI, general product identifier.

Supplemental Table 4 List of HCPCS codes for long-acting injectables

HCPCS	Description
C9470	Injection, aripiprazole lauroxil, 1 mg
J1631	Injection, haloperidol decanoate, per 50 mg
J1942	Injection, aripiprazole lauroxil, 1 mg
J2358	Injection, olanzapine, long-acting, 1 mg
J2426	Injection, paliperidone palmitate extended release, 1 mg
J2680	Injection, fluphenazine decanoate, up to 25 mg
J2794	Injection, risperidone, long-acting, 0.5 mg

HCPCS, Health Care Common Procedure Coding System.

Supplemental Table 5 Baseline characteristics for systolic and diastolic blood pressure cohorts

Characteristic	Systolic blood pressure analysis cohort[a] (n = 600)	Diastolic blood pressure analysis cohort[a] (n = 601)
**On-treatment period, mean (SD) [Q_1, median, Q_3]**, d	220 (186) [90, 153, 301]	220 (185) [90, 152, 300]
**Age, mean (SD)**, y	43.4 (13.1)	43.3 (13.1)
**Female,[b] n (%)**	450 (75.0)	451 (75.0)
**Weight, kg, mean (SD)**, kg	--	--
**BMI, mean (SD)**, kg/m^2	--	--
**Quan-CCI, mean (SD)**	0.69 (1.26)	0.69 (1.26)
**Race or ethnicity, n (%)**		
Black/African American	43 (7.2)	43 (7.2)
Hispanic	30 (5.0)	30 (5.0)
White	316 (52.7)	317 (52.7)
Other/Unknown[c]	14 (2.3)	14 (2.3)
Missing	197 (32.8)	197 (32.8)
**Geographic region, n (%)**		
Midwest	243 (40.5)	243 (40.4)
South	182 (30.3)	183 (30.4)
Northeast	122 (20.3)	122 (20.3)
West	52 (8.7)	52 (8.7)
Unknown	1 (0.2)	1 (0.2)
**BMI by category, n (%)**		
Underweight, BMI < 18.5 kg/m^2	--	--
Normal, BMI 18.5-25 kg/m^2	--	--
Overweight, BMI 25-30 kg/m^2	--	--
Obese, BMI ≥ 30 kg/m^2	--	--
**Prior medication use with risk of weight gain,[d] n (%)**		
Low risk of weight gain	372 (62.0)	372 (61.9)
Medium/high risk of weight gain	228 (38.0)	229 (38.1)
**Comorbidities, n (%)**		
Anxiety disorders	307 (51.2)	307 (51.1)
Sleep-wake disorders	173 (28.8)	173 (28.8)
Substance-related and addictive disorders	168 (28.0)	168 (28.0)
Hypertension	208 (34.7)	208 (34.6)

Diabetes	122 (20.3)	122 (20.3)
Drug abuse	96 (16.0)	96 (16.0)

[a]Reflective of the patient population with at least one measurement during the baseline period and at least one measurement during the on-treatment period.
[b]Information on patient sex was derived from Symphony Health Integrated Dataverse records.
[c]The term *other* stands for all races and ethnicities other than Black, Hispanic, and White.
[d]Medications with a low risk of weight gain included aripiprazole, ziprasidone, asenapine, brexpiprazole, lurasidone, paliperidone, typical antipsychotics, or no prior atypical antipsychotic during baseline. Medications with a medium/high risk of weight gain included clozapine, olanzapine, quetiapine, risperidone, or iloperidone during baseline. Patients with medication use defined as both low risk and medium/high risk were classified as medium/high risk (31).
BMI, body mass index; Quan-CCI, Quan-Charlson comorbidity index.
